# Bilirubin potentiates etomidate-induced sedation by enhancing GABA-induced currents after bile duct ligation

**DOI:** 10.1186/s40360-023-00675-w

**Published:** 2023-09-23

**Authors:** Hao Gao, Qian Zhao, Jian-Gang Song, Guo-Xia Hu, Wei-Feng Yu, Ying-Fu Jiao, Jin-Chao Song

**Affiliations:** 1 Department of Anesthesiology, Shidong Hospital of Shanghai, University of Shanghai for Science and Technology, Shanghai, China; 2 Department of Anesthesiology, Shanghai Shuguang Hospital, University of Traditional Chinese Medicine, Shanghai, China; 3 Department of Transfusion Medicine, Changhai Hospital, Naval Medical University, Shanghai, China; 4 Department of Anesthesiology, Renji Hospital Affiliated to School of Medicine, Shanghai Jiaotong University, Shanghai, China

**Keywords:** Bilirubin, Etomidate, Obstructive jaundice, GABA_A_ receptors

## Abstract

**Objectives:**

Our previous clinical trial showed that etomidate requirements to reach an appropriate level of anesthesia in patients with obstructive jaundice were reduced, which means that these patients are more sensitive to etomidate. However, the mechanism is still not completely clear. The present study was aimed to investigate the mechanism by which bilirubin facilitates etomidate induced sedation.

**Methods:**

A bile duct ligation (BDL) rat model was used to simulate obstructive jaundice. Anesthesia sensitivity to etomidate was determined by the time to loss of righting reflex (LORR). Intrathecal injection of bilirubin was used to test the effects of bilirubin on etomidate induced sedation. The modulating effects of bilirubin on GABA responses were studied using the whole-cell patch clamp technique.

**Results:**

The time to LORR induced by etomidate was significantly decreased in the BDL groups (*p* < 0.05), and unconjugated bilirubin in serum and cerebrospinal fluid (CSF) were markedly increased (*p* < 0.05). The time to LORR induced by etomidate was decreased after intrathecal injection of bilirubin (*p* < 0.05). A bilirubin concentration of 1.0 μM increased the GABA-induced currents of rat cortical pyramidal neurons (*p* < 0.05). Furthermore, 1.0 μM bilirubin enhanced GABA-induced currents modulated by etomidate (*p* < 0.05).

**Conclusions:**

Our results demonstrated that pathologic bilirubin in CSF could enhance etomidate induced sedation. The mechanism may be that bilirubin increase the GABA-induced currents of rat pyramidal neurons.

## Background

The incidence of hyperbilirubinemia is very high in China because of the high prevalence rate of hepatobiliary diseases [1]. Cholelithiasis, inflammation or tumors block the bile ducts and lead to obstructive jaundice [2, 3].

Patients with obstructive jaundice are observed to have concomitant higher pain thresholds, which might obscure clinical manifestations of the primary disease and delay hospitalization. Interestingly, although obstructive jaundice causes a numb sense of pain, patients are more sensitive to both inhaled and intravenous anesthetics [1, 4, 5] during the perioperative period and often suffer postponed wakening and respiratory depression after surgery. Thus, the amount of analgesic and sedative drugs has to be reduced in patients with obstructive jaundice.

Etomidate, a derivative of imidazole and an agonist of GABA_A_ receptors [6–8], is a commonly used intravenous anesthetic. Our previous study demonstrated that patients with obstructive jaundice had a greater sensitivity to etomidate, that etomidate requirements to reach a level of anesthesia defined by a bispectral index of 50 are reduced in patients with obstructive jaundice, and that there was a high negative correlation between serum total bilirubin and etomidate requirements [9]. However, the mechanism is still unclear.

Unconjugated bilirubin (i.e., indirect bilirubin, IB) can cross the blood–brain barrier (BBB) and induce a variety of biological effects that disturb the stabilization of the central nervous system (CNS) [10–12]. IB reduces neural oxygen consumption and increases the release of calcium, which influences neurotransmission [13]. Altered neurotransmission and neurotoxicity of bilirubin in the brain may partly contribute to the reduction in anesthetic requirements [14–16]. Some studies have investigated the effect of bilirubin on the mammalian CNS during general anesthesia [17–19].

Inhibitory GABA_A_ receptors, which are chloride-conducting pentameric ligand-gated ion channels, are the primary sites of action for etomidate [6–8]. In the present study, our aim was to test our hypothesis that pathologic bilirubin in CSF facilitates etomidate induced sedation by enhancing the GABA responses after bile duct ligation.

## Methods

### BDL models

Adult male Sprague‒Dawley rats (4–6 weeks old, weighing 200–250 g), were provided by the Experimental Animal Center of Shanghai Second Military Medical University. The use and handling of experimental animals were carried out in accordance with the relevant regulations of the State and the Experimental Animal Center of the Second Military Medical University. The rats were housed in cages (6 rats per cage) under a permanent temperature of 20–25 °C and a 12 h light/dark cycle. All the rats were allowed free access to food and water.

A bile duct ligation (BDL) rat model was used to simulate obstructive jaundice. The rats were randomly allocated to five experimental groups (6 rats/group). (1) control group; (2) sham group; (3) BDL-7 group; (4) BDL-14 group and (5) BDL-21 group. Rats were anesthetized by intraperitoneal administration of 60 mg/kg sodium pentobarbital (2%) before the operation. The anterior abdominal wall was disinfected with 10% povidone iodine standardized solution. Laparotomy was performed by making an incision on the midline of the abdominal skin and muscle. The common bile duct was exposed by gently separating the duodenum to the anteroinferior position. The common bile duct was ligated with 4–0 silk, and the abdomen was closed with an interrupted 3–0 silk suture. The difference between the sham-operated group and the BDL groups was whether the common bile duct was ligated. After the experiment, the rats were rapidly sacrificed by inhaling an overdose of sevoflurane.

### Behavioral tests on loss of righting reflex

Etomidate fat emulsion injection (2%, excipients: refined soybean oil, lecithin, glycerin, water for injection. Jiangsu Nhwa Pharmaceutical Co., Ltd.) was intravenously infused at a rate of 400 μg·kg^−1^·min^−1^ by a microinjection pump (Baoding Longer Precision Pump Co. Ltd) through a 24 G intravenous catheter system after the establishment of an effective and reliable tail venous channel. Rats were gently positioned on their back and their conscious state was observed every 20 s. When no righting attempts or movements in response to repeated positioning on their back were observed, the infusion was stopped. Anesthesia sensitivity to etomidate was evaluated by recording the latency of loss of righting reflex. The specified latency onset was after intravenous administrations and the termination was absence of the righting reflex continuing for 20 s. Rats were then gently prodded every 20 s to determine the onset of recovery, defined as recovery of the righting reflex. The investigators involved in behavioral tests on loss of righting reflex were unaware of the group allocation.

### Intrathecal injection of bilirubin

Rats were randomly divided into bilirubin groups (Bil-0.5, Bil-1.0, Bil-2.0), a vehicle group and a control group (*n* = 6 rats/group). Among these groups, the bilirubin group and vehicle group underwent surgical lateral ventricle catheterization. After a week of postoperative recovery to eliminate traumatic stress, we removed the needle stylets and inserted matching catheters to microinject bilirubin at different concentration (0.5 μM, 1.0 μM and 2.0 μM) in the bilirubin groups and CSF in the vechicle group. Twenty minutes later when rats were relieved from acute stress caused by intrathecal injection, etomidate (400 μg·kg^−1^·min^−1^) was intravenously infused and the time from the waking state to LORR and the time from LORR to regaining consciousness were recorded.

### Acute brain slice preparation

Newborn SD rats (P10-14) were provided by the Experimental Animal Center of Shanghai Second Military Medical University. The use and handling of experimental animals were carried out in accordance with the relevant regulations of the State and the Experimental Animal Center of the Second Military Medical University. Rats were anesthetized with sevoflurane. The brain was rapidly removed and immersed in ice-cold artificial cerebrospinal fluid (a-CSF), containing 124 mM NaCl, 26 mM NaHCO_3_, 1 mMNaH_2_PO_4_, 2.5 mM KCl, 2.5 mM CaCl_2_, 2 mM MgCl_2_, and 10 mM d-glucose, and bubbled with 95% O_2_/5% CO_2_ (pH 7.4). Slices were made using a Leica VT1200s vibro slicer, and incubated at 33 °C for one hour. Slices were maintained in the holding chamber at room temperature (23 °C) until required for electrophysiological analysis.

### Electrophysiology

Whole-cell currents were recorded by standard whole-cell, tight-seal recording techniques on the cortical pyramidal neurons. Patch pipettes were made from borosilicate glass capillaries and had a resistance of 4–8 MΩ when filled with the pipette solution. The pipette solution contained: 120 mM KCl, 1 mM MgCl_2_, 11 mM EGTA, 10 mM HEPES, 1 mM CaCl_2_, and 2 mM adenosine triphosphate, adjusted to pH 7.2 with 0.1 M NaOH. Brain slices were placed in a submersion chamber on an upright microscope and viewed with an Olympus 40X water-immersion objective with differential interference contrast and infrared optics. Slices were perfused with a-CSF at a rate of 7 ml/min at 23 °C. Drugs were added by a fast drug delivery system and perfused into the recording chamber at a rate of 15 ml/min. Currents were recorded using an Axopatch 200B amplifier, filtered at 2 kHz, digitized online at 10 kHz using an analog-to-digital converter and analyzed with pClamp 10.2 software (Molecular Devices, USA).

### Statistics

All variables were explored for normality and showed that they were normally distributed. Data are expressed as the means ± SDs or SEMs, and statistical analysis was performed using paired analysis of variance (ANOVA) with Tukey’s test as the post test, linear regression or t test, as appropriate. SPSS software, version 19.0, was used for all of the statistical analyses, and differences with a *P* value less than 0.05 were considered statistically significant (*p < 0.05).

## Results

### Obstructive jaundice potentiated etomidate induced sedation in rats

**Fig. 1:**
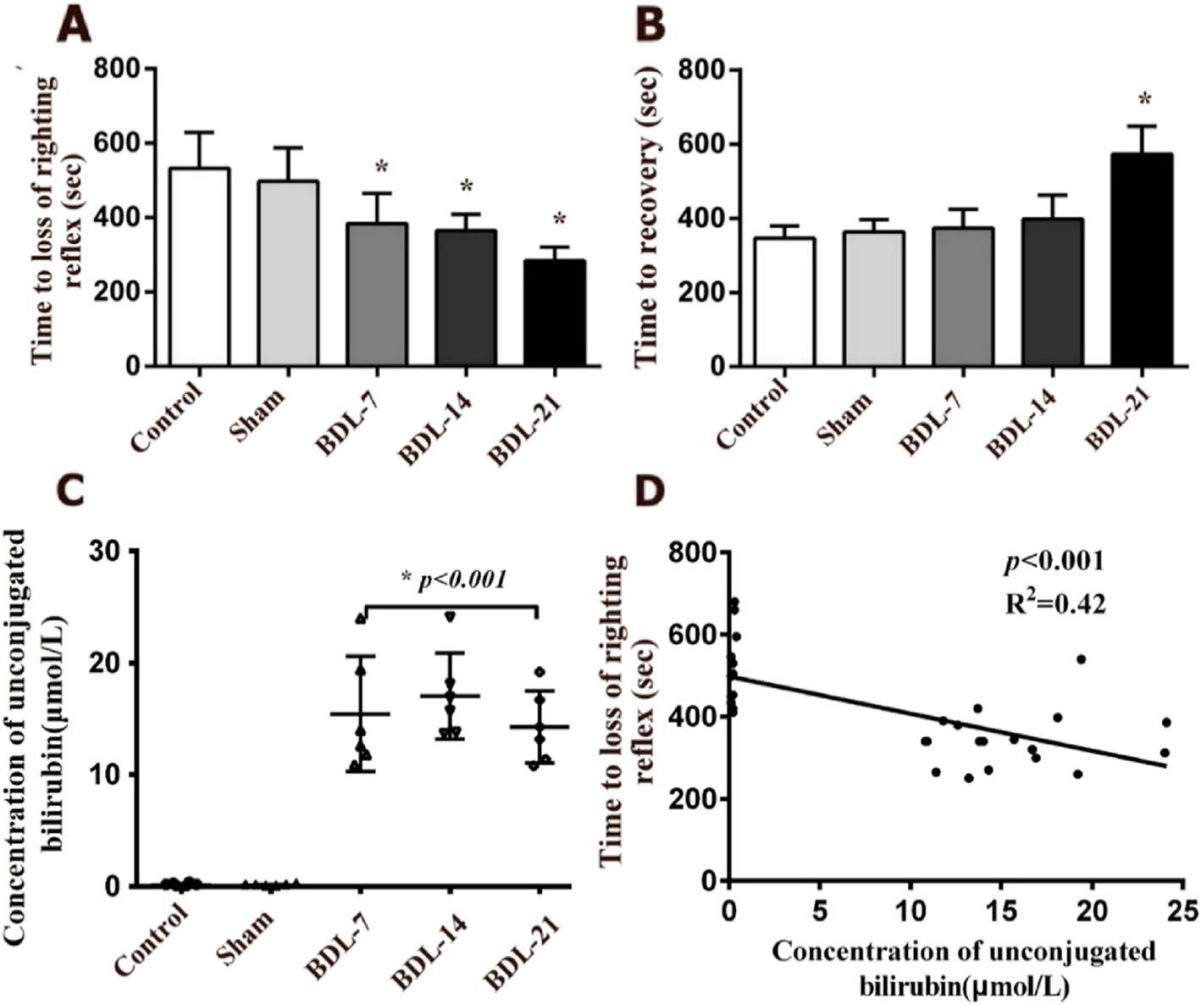
Obstructive jaundice potentiates the onset and duration of loss of righting reflex in rats. **A**, **B** Comparison of the times to loss of righting reflex and the time to recovery between four separate experiments (*n* = 6). **C** Serum unconjugated bilirubin concentration was measured. **D** Regression analysis between serum unconjugated bilirubin and times to loss of righting reflex. * *P* < 0.05, ANOVA

SD rats were divided into five groups: the control group (*n* = 6), sham group (*n* = 6), BDL-7 group (*n* = 6), BDL-14 group (*n* = 6) and BDL-21 group (*n* = 6). Compared with the control group, obstructive jaundice reduced the time to loss of righting reflex with etomidate administration from 532 ± 87 s to 284 ± 33 s (*p* = 0.014 BDL-7 vs. control; *p* = 0.005 BDL-14 vs. control; *p* < 0.001 BDL-21 vs. control, Fig. 1A). The time to recovery was also significantly increased for the BDL-21 group (*p* < 0.001 vs. control, Fig. 1B).

Because unconjugated bilirubin is toxic to the central nervous system, the concentrations of unconjugated bilirubin in serum were measured after the behavioral tests. Compared with that in the control group, the concentrations of serum unconjugated bilirubin in BDL groups increased significantly as hyperbilirubinemia (15.45 ± 4.71 μM for BDL-7, 17.05 ± 4.93 μM for BDL-14, and 14.27 ± 2.93 μM for BDL-21, vs. 0.22 ± 0.11 μM in the control group; *p* < 0.05, Fig. 1C). In addition, regression analysis reveals a significant correlation between serum unconjugated bilirubin concentration and the etomidate requirements (*P* < 0.05, *R*^*2*^ = 0.42, Fig. 1D).

### The concentration of unconjugated bilirubin in CSF increased after common bile duct ligation

**Fig. 2:**
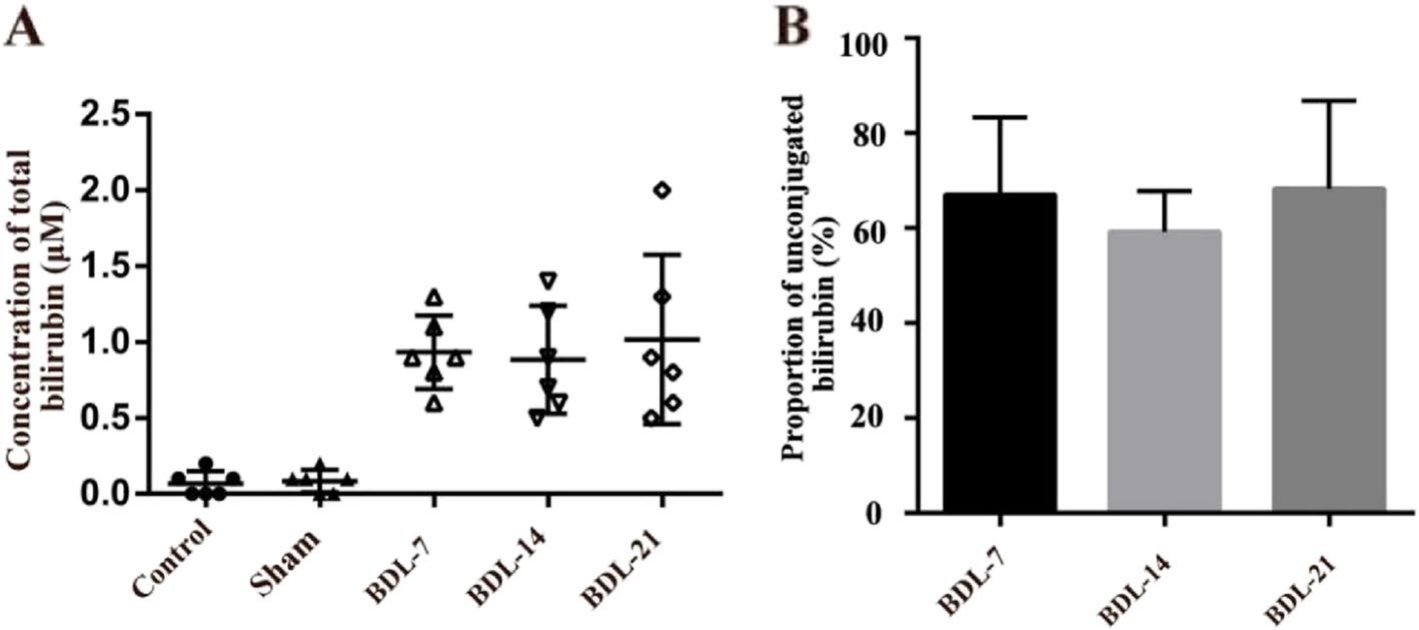
Concentration of bilirubin in rat cerebrospinal fluid (CSF) and proportion of unconjugated bilirubin. **A** Comparison of the concentration of bilirubin in rat CSF between five separate experiments (*n* = 6). **B** Proportion of unconjugated bilirubin in rat CSF

We simultaneously collected CSF from cisterna magna in the control group (*n* = 6), sham group (*n* = 6), BDL-7 group (*n* = 6), BDL-14 group (*n* = 6) and BDL-21 group (*n* = 6). The results showed that the concentration of bilirubin in CSF was almost zero in control or sham-operated rats, but was significantly increased in BDL groups (Fig. 2A).The proportion of unconjugated bilirubin in CSF among BDL rats was approximately 65% (50% -78%), while the concentration of conjugated bilirubin was several dozen times higher than that of unconjugated bilirubin in serum (Fig. 2B).

### Intrathecal injection of bilirubin enhanced rat sensitivity to etomidate

Considering the nonphysiological elevated levels of bilirubin in CSF after obstructive jaundice and the potential neurotoxicity of bilirubin, it is important to determine whether increased bilirubin in CSF could enhance the sensitivity to etomidate in rats.

**Fig. 3:**
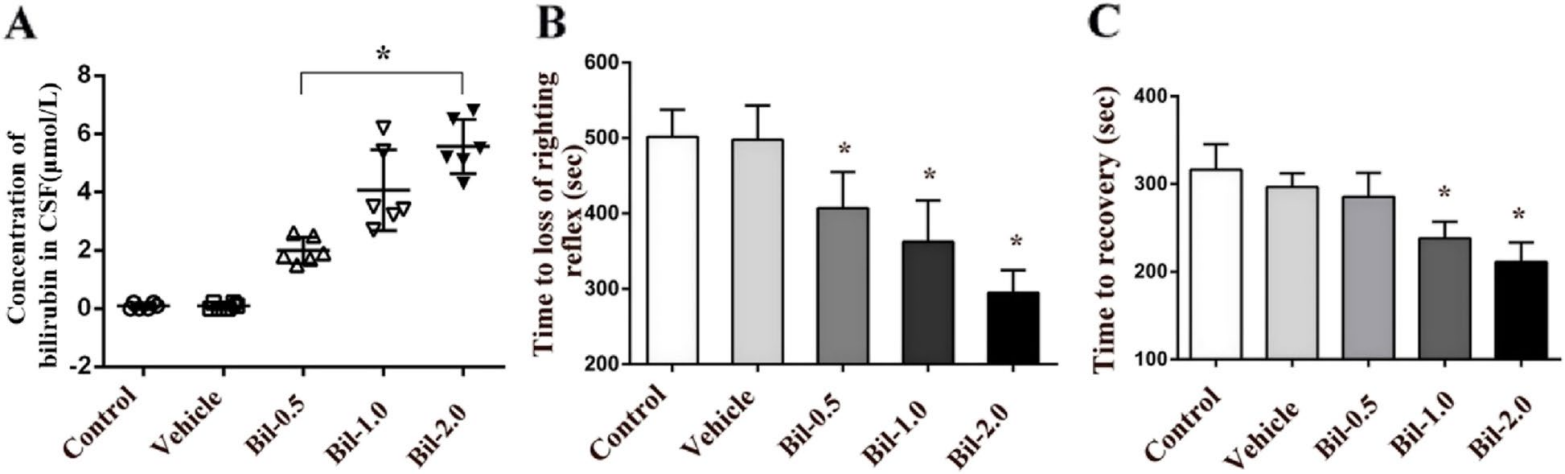
Intrathecal injection of bilirubin enhanced the sensitivity of rats to etomidate. (**A**) Concentration of bilirubin in CSF after intrathecal injection of different concentrations of bilirubin; (**B**, **C**) Comparison of times to loss of righting reflex and time to recovery after intrathecal injection of different concentrations ofbilirubin (*n* = 6). * *P* < 0.05, ANOVA

First, we measured the concentration of bilirubin in rat cerebrospinal fluid after lateral ventricle injection of bilirubin. We observed a direct and significant increase in the concentration of bilirubin in the cerebrospinal fluid (*P* < 0.05, Fig. 3A). Next, we examined the time to LORR and recovery time after lateral ventricle injection of bilirubin. Compared with the sham-operated control, lateral ventricle injection of bilirubin significantly reduced the time to LORR induced by etomidate (*P* < 0.05, Fig. 3B). The time to recovery was also significantly increased for the Bil-1.0 and Bil-2.0 groups (*p* < 0.05, Fig. 3C).

### Dose–response curve of GABA-evoked currents in rat pyramidal neurons

**Fig. 4:**
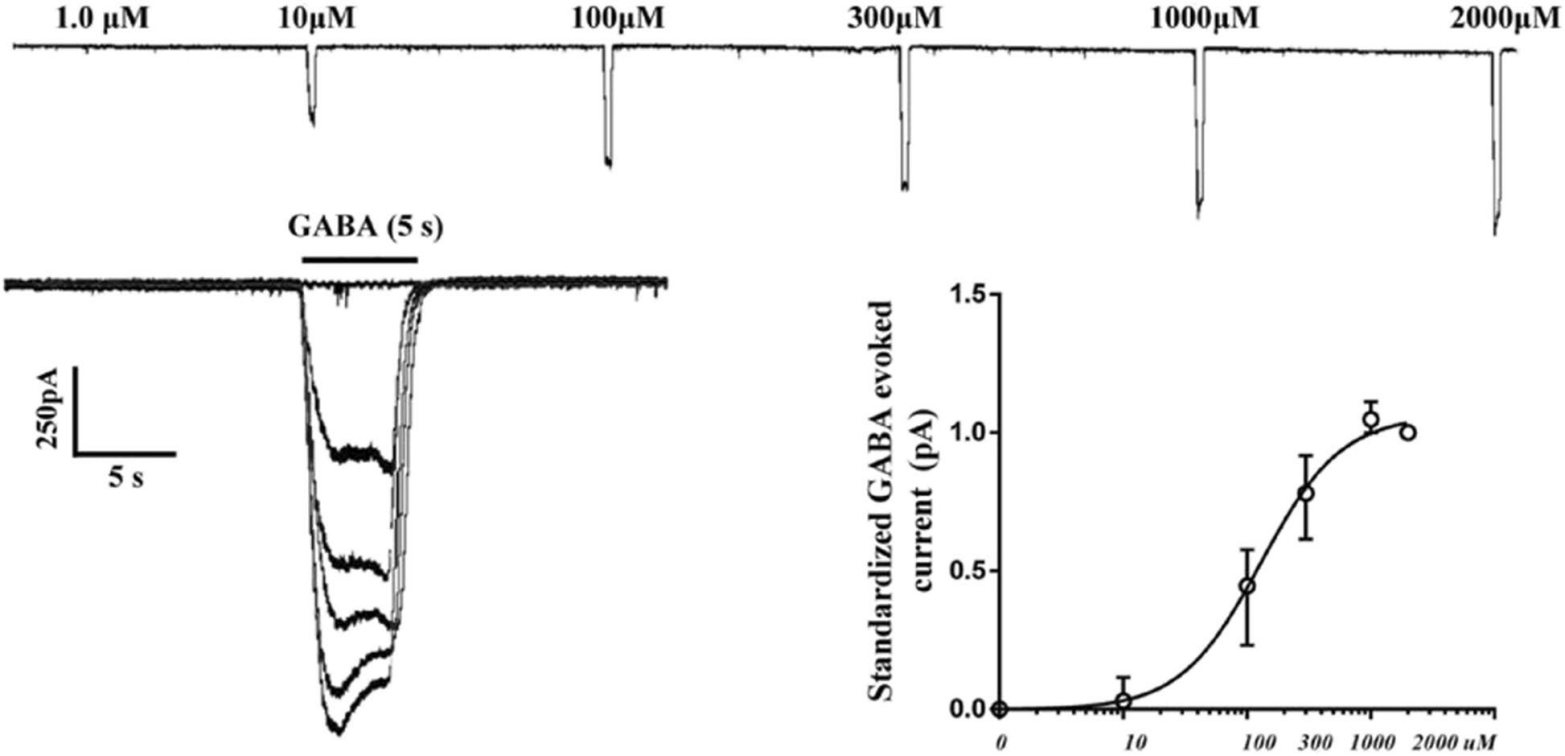
Dose–response curve of rat cortex pyramidal neurons GABA_A_Rs currents. **A** Representative GABA_A_Rs peak current traces evoked by 1, 10, 100, 300, 1000 and 2000 μM GABA. **B** Dose–response curve of GABA_A_Rs currents fitted by nonlinear regression with logistic equation. Each data point represents the mean ± SEM (*n* = 5)

The patch-clamp technique was applied to examine the response of the cortical pyramidal neurons to GABA or bilirubin. The cortical pyramidal neurons were identified based on their morphological characteristics. GABA could elicit a transient peak inward current at a holding potential of -60 mV. We recorded the peak amplitudes of the inward current response to 1, 10, 100, 1000 or 2000 μM GABA in cortical pyramidal neurons (Fig. 4A). The peak amplitudes of GABA-induced currents increase in a concentration-dependent manner and the maximum value was corresponding to 1000 μM GABA according to previous research [6]. In our experiment, the concentration for 50% of maximal effect (EC_50_) for GABA-dependent peak responses in cortical pyramidal neurons was 129.6 μM (95%CI, 99.97 μM ~ 168.0 μM) (Fig. 4B).

### Bilirubin enhanced the modulatory effect of etomidate on GABA responses

**Fig. 5:**
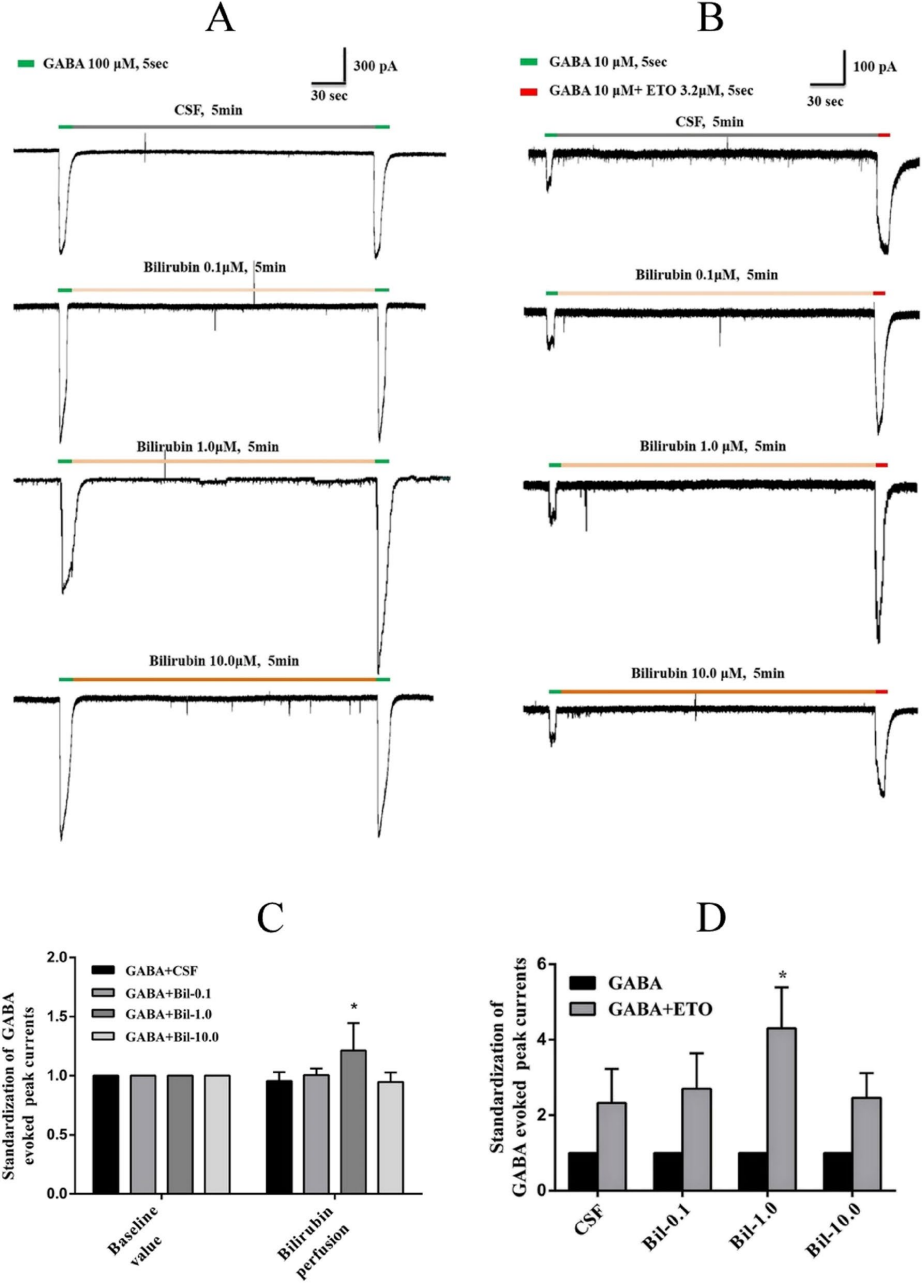
Bilirubin enhances the modulatory effect of etomidate on GABA responses. **A** Bilirubin (1.0 μM) enhanced GABA- induced inward currents. **B** Pretreatment with bilirubin (1.0 μM) before application of GABA (10 μM) further enhanced the effect of etomidate (3.2 μM) on GABA responses (*n* = 6). * *P* < 0.05

GABA_A_ receptors are the primary sites of action for etomidate, and are mainly located on the cell membrane of neurons. We further explored the effect of bilirubin on the GABA_A_ receptor. First, we applied different concentrations of bilirubin (0.1, 1.0, 10.0 μM) to observe the inward current changes of GABA_A_ receptor on pyramidal neurons. GABA (100 μM) induced inward currents were enhanced after the administration of 1.0 μM bilirubin (Fig. 5A, *P* < 0.001), but this phenomenon was not observed at lower (0.1 μM) or higher concentrations (10.0 μM) of bilirubin.

GABA is an agonist of the GABA_A_ receptor, and etomidate enhances GABA-induced currents. Whether bilirubin can further enhance GABA-induced currents in the presence of etomidate is unclear. We tested the effect of etomidate (3.2 μM) on GABA-induced currents and found that etomidate produced an ~ 2.5-fold (Fig. 5B) increase in the peak amplitude of GABA-induced currents when the concentration of GABA was 10.0 μM. Subsequently, different concentrations of bilirubin (0.1 μΜ, 1.0 μM or 10.0 μM) were introduced before application of GABA and etomidate. We observed that 1.0 μM bilirubin, which was close to the concentration detected in in the cerebrospinal fluid of BDL rats, futher enhanced GABA-induced currents in the presence of etomidate.

## Discussion

The aim of this study was to examine whether morbid bilirubin caused by obstructive jaundice could potentiate etomidate induced sedation. The results showed that BDL rats were more sensitive to etomidate in that unconjugated bilirubin in CSF could increase the GABA-induced currents of cortical pyramidal neurons.

Exorbitant levels of serum bilirubin clinically indicate the possibilities of liver dysfunctions, bile duct obstruction or hemolytic diseases. Unconjugated bilirubin is permeable to the BBB without liver transformation and can inhibit the oxygen metabolism of neurons [13]. Considerable efforts have been made to characterize the molecular, biochemical, and cellular mechanisms of bilirubin neurotoxicity [20]. Patients with hyperbilirubinemia are usually more sensitive to general anesthetics including narcotic analgesics and sedatives than normal patients [1, 4, 5]. We previously reported a clinical observation that patients with obstructive jaundice required a lower dosage of etomidate to reach sedation (BIS of 50) and etomidate consumption was inversely correlated with the severity of hyperbilirubinemia among this population during total intravenous anesthesia [9]. Etomidate was chosen in this study because it mainly acts on the GABA_A_ receptor which simplifies the follow-up research of the specific molecular mechanism [21–23].

We generated a BDL model in rats to mimic clinical cholestasis and tested whether acute jaundice could potentiate etomidate induced sedation. BDL rats were characterized by yellow mucosa and sclera, loss of appetite and clay colored stools. Cholestasis was objectively confirmed by proximal dilation of the common bile duct in autopsy. The BDL operation caused hyperbilirubinemia and BDL rats became more sensitive to etomidate over time. The time to LORR was negatively correlated with incremental concentrations of unconjugated bilirubin. The prolonged time to recovery in the BDL-21 group implicated the risk of postponed wakening in patients with hyperbilirubinemia. No significant prolongation of time to recovery was observed in the other BDL groups, which may be related to the lower dose of etomidate. These data suggested that unconjugated bilirubin may play an important role in hypersensitivity to etomidate in BDL rats.

LORR, as a surrogate for loss of consciousness (LOC), has long been used to effectively measure the depth of anesthesia in animals because unconsciousness is the most recognizable feature of general anesthesia [24, 25]. One of the underlying indices of LORR is the time from the start of drug administration to the loss of consciousness in rats. Since the etomidate-relevant latency from the waking state to LORR shortens sharply in BDL rats compared with that in the sham/control groups, we speculate that hyperbilirubinemia caused by ligation of the bile duct could potentiate etomidate induced sedation. Our findings may have implications for the clinical dose selection of anesthetics in patients with jaundice.

Unconjugated bilirubin is produced without liver transformation and is permeable to the BBB. A high of unconjugated bilirubin can be detected after the BDL operation in blood-free CSF (100–200 μl) by radioimmunoassay (RIA) with high sensitivity as long as the concentration of bilirubin exceeds 0.10 μM [26]. CSF plays a crucial role in CNS homeostasis and can be collected percutaneously from the cisterna magna under a stereotaxic frame in rats. Our results demonstrated that the concentration of bilirubin in the CSF of BDL rats significantly increased and was dominated by elevated unconjugated bilirubin (65%, 50%-78%). Hyperbilirubinemia and unconjugated bilirubin in CSF have been described as toxic to the brain, which may result in hypersensitivity to etomidate.

We attempted to verify this speculation by implanting cannulas into the rat lateral ventricle to conduct intrathecal injection of bilirubin. The etomidate-relevant latency of LORR was tested as described above after the dissolved bilirubin solution was injected through the cannulas. Interestingly, intrathecally administered bilirubin shortened the etomidate-relevant latency from the waking state to LORR. The results confirmed the conclusion that an abnormally increased bilirubin in the CNS could potentiate etomidate induced sedation.

A previous study by Brito MA et al*.* demonstrated that 10 μM bilirubin does not modulate ionotropic glutamate receptors or glutamate transporters [10] and has no effect on glycine-evoked postsynaptic currents. On the other hand, other studies hav reported that bilirubin could enhance GABA/glycinergic synaptic transmission in lateral superior olivary nucleus neurons [27] and modulaten AChRs in rat SCG neurons bidirectionally [28]. In the present study, we observed that 1.0 μM bilirubin applied for 5 min enhanced GABA (100 μM)-induced inward currents. Interestingly, the concentration of bilirubin was very close to the concentration that we detected in the CSF of the BDL groups. Pretreatment with 1.0 μM bilirubin before application of 10 μM GABA further enhanced the modulatory effect of etomidate (3.2 μM) on GABA-induced inward currents.

## Conclusion

In conclusion, we found that the BDL operation remarkably shortened the latency of LORR induced by etomidate and that exposure of neurons to CSF containing bilirubin enhanced GABA-induced currents with or without etomidate. Thus, we demonstrate that unconjugated bilirubin in the CNS could potentiate sensitivity to etomidate in rats with obstructive jaundice, and the mechanism may be that bilirubin can increase the GABA-induced currents of rat pyramidal neurons.

We explored the mechanism of hypersensitivity to etomidate in patients with obstructive jaundice. As bilirubin is pathological and anesthetics are a therapeutic interference with the normal internal environment of the brain, determining out how bilirubin and anesthetics interact with neurons can help provide guidance for accurate anesthetic use in hyperbilirubinemia and be an enlightening topic of neuroscience.

## Data Availability

The datasets used and/or analyzed during the current study are available from corresponding author upon reasonable request.
